# Antioxidants in Photoaging: From Molecular Insights to Clinical Applications

**DOI:** 10.3390/ijms25042403

**Published:** 2024-02-18

**Authors:** María José Calvo, Carolina Navarro, Pablo Durán, Nataly J. Galan-Freyle, Luis Alberto Parra Hernández, Leonardo C Pacheco-Londoño, Desiree Castelanich, Valmore Bermúdez, Maricarmen Chacin

**Affiliations:** 1 Endocrine and Metabolic Diseases Research Center, School of Medicine, University of Zulia, Maracaibo 4001, Venezuelacnm10698@gmail.com (C.N.); pabloduran1998@gmail.com (P.D.); 2 Centro de Investigaciones en Ciencias de la Vida (CICV), Universidad Simón Bolívar, Barranquilla 080003, Colombia; nataly.galan@unisimon.edu.co (N.J.G.-F.); leonardo.pacheco@unisimon.edu.co (L.C.P.-L.); valmore.bermudez@unisimon.edu.co (V.B.); 3 International Society for Non-Surgical Facial Rejuvenation (SIRF), Barranquilla 080003, Colombia; gerencia@drluisalbertoparra.com (L.A.P.H.); dgc1992@gmail.com (D.C.); 4 Argentine Society of Dermatology, Buenos Aires B1228, Argentina; 5 Facultad de Ciencias de la Salud, Universidad Simón Bolívar, Barranquilla 080003, Colombia

**Keywords:** photoaging, skin, DNA, antioxidant, nutraceutical

## Abstract

Photoaging (PA) is considered a silent disease affecting millions of people globally and is defined as skin damage due to prolonged exposure to ultraviolet radiation (UVR) from the sun. Physiologically, the skin is in a state of renewal and synthesis of components of the extracellular matrix (ECM). However, exposure to UVR affects the production of the ECM, and the functioning and response of skin cells to UVR begins to change, thus expressing clinical and phenotypic characteristics of PA. The primary mechanisms involved in PA are direct damage to the DNA of skin cells, increases in oxidative stress, the activation of cell signaling pathways responsible for the loss of skin integrity, and cytotoxicity. The medical and scientific community has been researching new therapeutic tools that counteract PA, considering that the damage caused by UVR exceeds the antioxidant defense mechanisms of the skin. Thus, in recent years, certain nutraceuticals and phytochemicals have been found to exhibit potential antioxidant and photoprotective effects. Therefore, the main objective of this review is to elucidate the molecular bases of PA and the latest pharmaceutical industry findings on antioxidant treatment against the progression of PA.

## 1. Introduction

The skin is a specialized, large, and multifunctional organ that covers the entire body, prevents dehydration due to the evaporation of body fluids, and participates in thermoregulation. It also plays an important role in the immune system as a protective barrier. As the outermost layer, the skin is the organ most exposed to the environment and pollutants [1]. With the passage of time, similar to the rest of the organs, the skin is affected by chronological aging (CA), which involves the accumulation of lesions and the deterioration of physiological processes ranging from purely molecular to macroscopic levels. Therefore, CA affects the function and architecture of the skin [2].

In addition to CA, photoaging (PA) plays a major role in skin aging. PA is caused by prolonged exposure to the sun’s ultraviolet radiation (UVR), which—in synergy with other environmental factors—promotes the production of reactive oxygen species (ROS), leading to oxidative stress (OS) and the activation of molecular pathways that directly or indirectly damage the DNA [3,4]. Furthermore, UVR can disrupt the integrity of different skin layers and promote a proinflammatory state that induces skin damage. These disruptions result in deep wrinkles, solar elastosis, hyperpigmented spots, telangiectasia, or even the progression of precancerous lesions [5,6]. The severity of the development of PA can be reflected in the appearance of premature aging even in 30-year-old individuals [7].

Thus, UVR is considered the basis of PA. Clinical manifestations of PA predominate the skin sites most exposed to the sun, such as the face, neck, chest, forearms, and hands, and mainly affect people with a light-colored skin phototype compared with those with darker skin [8]. Furthermore, genetic factors, lifestyle, ethnicity, and other sociodemographic characteristics can predispose a person to the progression of CA and PA. For example, UVR levels are high in low-latitude tropical areas and affect 80% of the world’s population [9]. In addition, smoking, frequent tanning habits, or occupations that demand prolonged exposure to the sun aggravate and accelerate the skin damage caused by PA [10].

Therefore, the scientific and cosmetological communities have focused on the development and research of therapeutic tools to reduce the deleterious effects of UVR. The tools include sunscreens, physical activity, and primarily the use of antioxidants, whose exogenous administration has been shown to prevent the effects of PA [11,12]. Thus, the aim of this review is to elucidate the molecular bases of PA and the latest findings in the pharmaceutical industry regarding antioxidant treatment against the progression of PA.

## 2. Materials and Methods

This review provides novel information on the function of antioxidants against PA; the literature review was not systematic. An extensive literature search was performed on Scopus, EMBASE, PubMed, ISI Web of Science, ScienceDirect, Medline, Cochrane Library Plus, and Google Scholar databases, from inception to December 2023. The articles used in this review were only those in Spanish and English. No restrictions were made according to the type of study. Scientific articles belonging to high-impact journals were selected: Q1, Q2, and Q3. The terms “Photoaging”, “Antioxidant”, “ROS”, “DNA damage”, and “oxidative stress” were among the ones used throughout the search.

## 3. Physiological Aging: The Skin’s Stopwatch

Intrinsic or CA of the skin refers to the deleterious mechanisms that occur over time in the three layers of this tissue. Several theories attempt to explain the advancement of aging. One of the most notable theories states that this process is a consequence of multiple damages that accumulate at the intra- and extracellular levels, including damage to the genome or epigenome, telomere shortening, loss of proteostasis, and even mitochondrial dysfunction. These disturbances do not happen independently, but rather converge and lead to an increase in the production of ROS, cell cycle arrest or senescence, and the dysregulation of nutrient uptake. This causes a depletion in the number of available stem cells and a dysfunction in intercellular communication [13,14]. Conversely, other researchers have associated the CA process and the aforementioned mechanisms with the overexpression of insulin-like growth factor 1 (IGF1) [15].

One of the molecular pathways associated with the development of CA is the change in transforming growth factor β (TGFβ), a pleiotropic cytokine. TGFβ stimulates dermal fibroblasts, which are responsible for synthesizing type I collagen, elastin, and glycosaminoglycans (GAGs), all components of the extracellular matrix (ECM) that give the skin its elastic properties. On the other hand, TGFβ can regulate and reduce the production of endopeptidases, such as matrix metalloproteinases (MMPs), which degrade the collagen and elastin fibers of the ECM [16,17].

**Figure 1:**
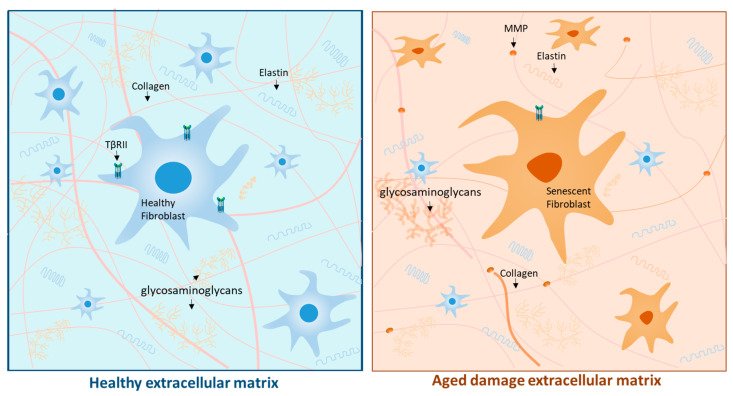
Physiological Aging. As we age, the skin’s extracellular matrix (ECM) undergoes a progressive deterioration due to various mechanisms, leading to the manifestation of fragile and thin skin, along with multiple wrinkles. One of the mechanisms responsible for this is the downregulation of TβRII, which leads to a decrease in ECM components and increased synthesis of MMPs. Abbreviations: ECM: extracellular matrix; TβRII: transforming growth factor β receptors; MMPs: metalloproteinases.

Fibroblasts lose their original morphology during CA, compromising TGFβ signaling through the downregulation of the TGFβ type II receptor (TβRII). Consequently, these changes lead to a decrease in the synthesis of the structural components of the ECM [18]. In addition, alterations in TGFβ signaling correlate with an increase in the production of MMPs, particularly MMP1 and MMP3, as shown in Figure 1 [19]. On the other hand, changes related to CA lead to the senescence of fibroblasts. In this context, cellular senescence refers to the process in which the cell exhausts its proliferative capacity (due to the shortening of telomeres) and, thus, halts its cell cycle, entering a state of latency. This process can also occur as a response of the cell to intracellular damage to prevent the formation of tumors [20,21].

Senescent fibroblasts remain metabolically active and must be removed by the immune system because they begin to develop a senescence-associated secretory phenotype (SASP) over time, secreting cytokines, chemokines, and MMPs, which increase ECM degradation. Moreover, SASP can transform healthy neighboring cells into senescent cells, further propagating its deleterious effects in the tissue [22]. These phenomena, together with the activation of multiple deleterious pathways of the immune system, are currently called “inflammaging,” which refers to the changes mediated by chronic inflammation combined with aging [23].

Consequently, alterations in the secretome of fibroblasts could be responsible for the exhaustion and dysfunction of epidermal stem cells and, therefore, cause a decrease in the number of keratinocytes in the elderly [24]. These changes are observed macroscopically as rigid, fragile, and thin skin with the appearance of fine wrinkles due to the deterioration and cross-linking of collagen, which is accompanied by skin dryness due to the water loss caused by the decrease in GAGs of the skin’s ECM [25].

Thus, CA gradually deteriorates all the structures and functions of the skin, resulting in a decrease in its wound-repair capacity and immune response. It can also affect the functioning of sweat glands and subcutaneous adipose tissue, thus altering thermoregulation and many other physiological mechanisms [26]. The clinical manifestations of CA are essentially observed in those parts of the body that are most protected from the sun, such as the buttocks or the inner part of the arms, as areas such as the face, neck, and shoulders are constantly exposed to UVR and are more affected by PA, thus aggravating the manifestations of aging in the skin regardless of a person’s age [27].

## 4. Photoaging: Underlying Mechanisms and Implications of Oxidative Stress

The pathophysiological mechanisms of PA are mainly based on the production of ROS and the subsequent development of OS. ROS are free radicals caused by the unpairing of electrons in one of their layers and are characterized by being biochemically reactive and by trying to stabilize themselves by binding to other molecules [28]. The most common ROS are the superoxide anion (O_2_^•−^), hydroxyl radical (HO^•−^), hydrogen peroxide (H_2_O_2_), and singlet oxygen (^1^O_2_) [29].

Under various physiological states, these molecules act as second messengers to activate or modulate cell signaling pathways involved in cell growth, differentiation, progression, or death, and are released by immune cells, such as neutrophils, to combat microorganisms [30]. In addition, free radicals are in constant interaction with antioxidant molecules that catalyze redox reactions to keep the physiological levels of ROS in balance. However, sometimes certain exogenous stimuli, such as UVR, increase the generation of ROS, surpassing the body’s antioxidant capacity and leading to an inadequate response and alteration of homeostasis known as OS [31].

### 4.1. Characteristics of Ultraviolet Radiation

The production of ROS in PA, which are the primary factors in its pathophysiology, is mediated by prolonged exposure to UVR [32]. The important role of UVR lies in its mutagenic properties, as it can cause direct DNA damage, triggering the dysregulated activity of different molecular pathways, altering the lipid membrane structure, and affecting protein homeostasis, thus leading to pathological states of chronic skin inflammation [33].

Under this premise, UVR can be classified according to wavelength as follows: UV-C (100–280 nm), UV-B (280–320 nm), and UV-A (320–400 nm). The ozone layer filters the UV-C radiation and most of the UV-B radiation. Therefore, the types of UVR that reach the earth’s surface are UV-A (90–95%) and UV-B (5–10%) [34]. UV-B photons have higher energy than UV-A photons [35]. Because of their wavelength, UV-B radiation is able to irradiate only the epidermis and a very superficial part of the dermis, while UV-A radiation has a higher pathogenicity on the dermis [36].

The degree of PA and its clinical manifestations in each person depend on multiple factors, which are related to lifestyle, the degree of prevention and protection against UVR, and/or the person’s geographical location, as UVR is more energetic closer to the equator [37]. Similarly, the skin phototype of individuals is essential in determining the risk of PA, as it has been shown that those with light skin colors tend to be more prone to UVR damage than those with dark skin, possibly due to the proportion of melanocytes in the skin and their protective effects [35,38].

### 4.2. DNA Damage due to Ultraviolet Radiation

It has been widely reported in the literature that exposure to UVR causes numerous forms of damage at the cellular level, primarily in skin cells. One of the most serious forms of damage occurs at the DNA level, since damage is generated through different pathways and molecules that depend on wavelength and exposure time, leading to structural and functional alterations [39]. These alterations are responsible for both photoaging processes as well as multiple types of skin cancer due to said mutations [40].

UVB radiation is responsible for damaging DNA directly. It is absorbed more easily than UVA radiation [41]. When UVB radiation affects the DNA molecule, it generates excited states that produce three main compounds by creating covalent bonds in adjacent pyrimidine bases. The most common compound is the cyclobutane pyrimidine dimer (CPD), followed by pyrimidine 6-4 photoproducts of pyrimidone (6-4PPs), and its Dewar isomers (in smaller amounts) [42]. Among these compounds, CPDs are the most abundant and probably the most harmful. However, it has been reported that 6-4PPs are the most associated with mutagenesis processes, making them more lethal and severe [43].

Although the most prominent lesions correspond to pyrimidine bases, purine bases can also suffer damage to their structure due to UVB exposure [44] and, if not repaired, contribute to mutagenesis processes. Among the notable photoproducts generated in this pathway are adenine dimers and adenine–thiamine adducts [16].

On the other hand, UVA causes indirect damage to DNA. This is mainly produced by the oxidation of nitrogenous bases. One of the ways by which this phenomenon occurs is through the production of ROS from other types of chromophores such as riboflavin, which enters an excited state, changing its organization and promoting the formation of ¹O₂ from molecular oxygen [45,46]. Subsequently, these end up oxidizing bases such as guanine, converting it into 8-hydroxy-2′-deoxyguanine (8OHdG) [39,47].

Therefore, there are multiple mechanisms that can affect the DNA of the various cells that make up the skin. However, negative effects may vary in intensity depending on the type of cell. For instance, it is known that the oxidative impact of UVA radiation is more pronounced on melanocytes than on keratinocytes [48]. This means that direct or indirect DNA damage can lead to functional alterations in skin cells, such as changes in base pairing, DNA replication, transcription, breakage of one or both DNA strands, and the activation of pathways for skin repair, cell growth, and differentiation [49].

### 4.3. Reactive Oxygen Species and Cellular Signaling Pathways Responsible for the Loss of Skin Integrity

Another harmful effect of ROS on the skin is their ability to degrade collagen, the primary protein in the ECM of the dermis synthesized by fibroblasts, thus promoting the development of thick wrinkles and dry, lax-textured skin [50]. This is mediated by the activation of the mitogen-activated protein kinase (MAPK) pathway, which phosphorylates and elicits the activity of pathways such as JNK, P38 MAPK, and extracellular signal-regulated kinase (ERK). Thus, there is an increased expression of final products responsible for MMP transcription, such as transcription factor activator protein 1 (AP1) [51].

It has also been shown that UV-B radiation is mostly responsible for increasing the synthesis of collagenase 1 (MMP1) and stromelysin-1 (MMP3), while the increase in the expression of macrophage elastase (MMP12) is mediated by high exposure to UV-A radiation [52].

**Figure 2:**
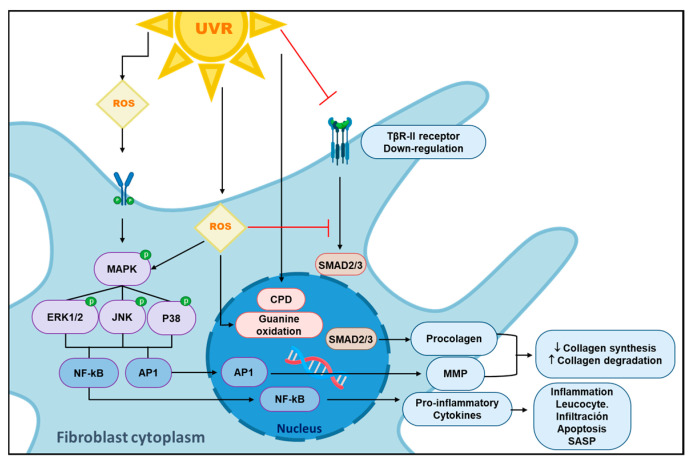
Fibroblast changes induced by ultraviolet radiation. Ultraviolet radiation (UVR) generates reactive oxygen species (ROS) in the intracellular and extracellular space, leading to the activation of MAPK signaling pathways. These pathways lead to transcription factors AP1 and NFκB translocating to the nucleus, and consequently, the transcription of metalloproteinases (MMPs) and proinflammatory cytokine (IL-1, IL-6, IL-8, TNFα) genes, all of which play pivotal roles in skin damage. Additionally, MMP synthesis can be mediated by TβRII downregulation. The effects of ROS also encompass plasma membrane lipoperoxidation, proteostasis disruption, and direct or indirect DNA damage. Abbreviations: UVR: ultraviolet radiation; ROS: reactive oxygen species; MAPK: mitogen-activated protein kinase; JNKs: c-Jun N-terminal kinases; P38: mitogen-activated protein kinase p38; NFκB: nuclear factor kappa beta; MMPs: matrix metalloproteinases; TβRII: transforming growth factor β receptor II; SASP: senescence-associated secretory phenotype; CPD: cyclobutane pyrimidine dimers.

In addition to the degradation of collagen, ROS also promote a decrease in the synthesis of this protein by impairing the TGFβ signaling pathway through the downregulation of TβRII. This causes a decrease in the phosphorylation and activation of the transcription factors (Smad 2/3) required for the transcription of type 1 procollagen, as shown in Figure 2 [53].

### 4.4. Ultraviolet Radiation-Mediated Cytotoxicity

Mitochondria are one of the main organelles whose dysfunction is widely related to aging and chronic diseases. These organelles are responsible for generating energy in the form of ATP necessary for the functioning of cells [54]. In recent decades, the participation of mitochondria in aging and chronic diseases has been widely studied and controversial. There are multiple theories that infer about the possible pathological pathways in which mitochondrial dysfunction participates; one of them is the so-called mitochondrial free-radical theory of aging [55].

Mitochondria are the primary source of ROS in cells because they are produced as byproducts during the metabolism of aerobic cells through the respiratory chain [56]. Under certain prolonged and pathological conditions, such as exposure to UVR rays, the production of ROS increases initially as a cellular defense mechanism. However, if this stimulus continues for an extended period, it leads to an excess of ROS [57], causing an imbalance in the mitochondrial respiration chain, leading to a further increase in ROS. This overproduction of ROS activates MAPK, which intensifies OS.

These factors work together to promote the deregulation of various mechanisms associated with inflammation, apoptosis, or necrosis processes. One of the most well-known mechanisms in PE is the translocation of nuclear transcription factor kappa B (NFκB) to the nucleus in both keratinocytes [1] and fibroblasts [58,59,60]. Once NFκB is in the nucleus, it increases the transcription of genes that stimulate the synthesis of proinflammatory cytokines such as interleukins (IL1β, IL6, IL8) and TNFα. This group of cytokines exacerbates oxidative-stress-mediated damage and leads to a vicious inflammatory and harmful cycle, since they provoke or cause a greater activation of MAPK and NFκB [61]. Similarly, this process is also associated with greater mitochondrial DNA damage and mutation which, to a certain extent, represents another point in the pathophysiology of CA [62].

Inflammation and cytotoxicity caused by exposure to UVR are major factors in the development of PA. If the damage to cells caused by the excessive activation of signaling pathways exceeds the repair mechanisms, it may lead to cell death or senescence, as well as processes related to autophagy [63].

Senescence, which is an active state of metabolism, is intensified by the effects of UVR. In this state, a senescence-associated secretory phenotype (SASP) is produced by the cells, which releases harmful products such as proinflammatory cytokines, chemokines, and proteases that damage tissues [16].

Autophagy, on the other hand, is a cellular homeostasis process that eliminates harmful intracellular materials like proteins and lipids [64,65]. It has been suggested as a protective mechanism against photoaging, but its relationship with UVR and the way it is activated needs further study. Moreover, the dysfunction of mitochondria can reduce the effectiveness of this mechanism [66].

## 5. Antioxidant System and Skin Health

The skin contains a complex antioxidant system capable of maintaining the redox homeostasis of ROS so that they do not destabilize other cellular molecules in the tissue [67]. There are different antioxidant molecules that act at different cellular levels, which can be divided into enzymatic and nonenzymatic factors of endogenous or exogenous origin [68].

Among the enzymes of endogenous origin that make up the antioxidant system are superoxide dismutase (SOD), catalase, and glutathione peroxidase, whose function is to catalyze the reaction that inactivates ROS, converting them mostly into less reactive species, such as water and/or oxygen. Among the factors of exogenous origin are those from foods, plants, and fruits, such as polyphenols (chlorogenic acid, gallic acid, and caffeic acid), flavonoids (quercetin, rutin, lutein, and resveratrol), vitamins (A, C, and E), and minerals (potassium, magnesium, iron, and sodium). The activity of nonenzymatic antioxidants is diverse, as they can behave as electron acceptors or donors, increase the expression of genes associated with the production of antioxidant enzymes, inhibit pro-oxidant enzymes, or act as enzymatic cofactors (minerals) [69,70,71].

Research on the properties of the antioxidant molecules present in multiple natural compounds, plants, and fruits, has currently gained substantial attention in the scientific community. Research on the functions, pathways, and mechanisms through which these antioxidants act has been particularly encouraged. In the field of dermatology, most studies have provided satisfactory data for the preventive treatment of PA, as the exogenous antioxidant compounds present in natural products show positive effects against the pathogenic processes of PA. This applies whether the compounds are administered through a diet based on foods rich in antioxidants [72], dietary supplements containing isolated antioxidant molecules [73], or topically, by including antioxidant-rich products in skincare routines [74].

## 6. Antioxidants against Photoaging: Clinical Evidence

For many years, the agents used against PA have been based on two principles: (1) preventing the effects of UVR on the skin and (2) addressing the damage they cause [75]. Regarding the first principle, the strategies of choice are photoprotection through the use of sunscreens, wearing protective clothing to shield the skin from UVR (organic filters), and avoiding peak sun exposure hours. For addressing the damage already caused to the skin, topical retinoids, such as tretinoin and tazarotene (vitamin A derivatives), are the only medications approved by the FDA (Food and Drug Administration) for the treatment of PA. However, scientific discoveries have led to the development of a new therapeutic tool: antioxidants from nutritional supplements and medicinal plants (summarized in Table 1 [76,77]).

In this regard, vitamin C is one of the main antioxidants present in various nutritional supplements and fruits, such as orange and lemon. Vitamin C counteracts lipid peroxidation, reduces ROS-induced damage, inhibits NfκB activation, stimulates collagen production by enhancing its genetic expression, and acts as a collagen-stabilizing molecule structure because of its action on lysine and proline hydroxylases [78,79,80]. Furthermore, a study on 33 patients using electron paramagnetic resonance spectroscopy showed that vitamin C supplementation at doses of 100 mg/d and 180 mg/d for 4 weeks caused an increase in ROS elimination by 22% and 37%, respectively [81]. However, some investigations have failed to show the role of orally administered vitamin C in PA [82,83].

Several clinical trials and in vivo studies have demonstrated that the topical use of vitamin C at different concentrations shows high efficacy in the treatment of skin damaged by PA, with evidence of its inhibitory effect on MMP-1 [84], a decrease in PA scores of the face, an increase in collagen production [85], a significant increase in cutaneous microrelief density, a reduction in deep wrinkles [86], the suppression of genetic mutations associated with UV exposure [87], and protection against erythema [88] and skin hyperpigmentation [89]. The joint use of vitamins C and E generates a synergistic effect on the antioxidant activity of both compounds. Placzek et al. [90] reported that the oral administration of a combination of vitamins C and E for 3 months in individuals with dermatological damage induced by UV-B radiation caused a significant decrease in thymine dimers, an indicator of DNA damage. These results were in agreement with those obtained by Murray et al. [87]. Furthermore, similar to vitamin C, oral supplementation of vitamin E alone leads to inconclusive or unpromising results [91].

Polyphenols from several medicinal plants and herbs have shown positive effects against PA. Antioxidant flavonoids and phenolic acids from *Opuntia ficus-indica*, *Zuccagnia punctata*, *Larrea divaricata*, *Larrea cuneifolia*, *Larrea nitida*, calendula, rosemary, açaí palm fruit, and grape seed extracts show protective mechanisms against UV-A and UV-B radiation, as they counteract the production of ROS and, therefore, OS. They are also capable of maintaining stable lipid peroxidation levels, inhibiting keratinocyte apoptosis, inhibiting collagen degradation, inhibiting MMP-1, reducing the inflammatory state associated with epithelial damage, and promoting the expression of key factors in cellular repair [92,93,94,95,96,97].

Resveratrol (RS) is a natural polyphenol with various benefits, including anti-aging, antioxidant, and anti-obesogenic effects. Its topical application has proven to be beneficial for the treatment of PA and CA [98,99]. According to Lephart et al. [100], the application of 1% RS can stimulate the genetic expression of sirtuin 1 (SIRT1), a protein known for its anti-aging effects. It can also modulate the apoptosis of keratinocytes after exposure to UVB. RS increases the concentrations of collagen types I and III, and elastin, both directly by increasing their synthesis, and indirectly by increasing the genetic expression of the tissue inhibitor of matrix metalloproteinases (TIMP 1), which leads to a decrease in MMP1 and MMP9, thereby reducing the rupture of collagen fibers. In addition, RS increases the expression of antioxidant enzymes, decreases the expression of proinflammatory cytokines, and increases the expression of anti-aging biomarkers such as proliferating cell nuclear antigen (PCNA) and nerve growth factor (NGF).

It should be noted that RS is most effective when used topically in the stratum corneum of the epidermis, where it generates greater thickness, better appearance, greater free-radical-scavenging capacity, and protection against UVR [101]. However, multiple studies show the benefits of RS when ingested orally in different forms [102,103].

In this regard, using immunohistochemistry and DNA microarray analysis, it has been reported that the topical application of green tea extracts rich in polyphenols at low concentrations significantly reduce p53 expression and the number of apoptotic keratinocytes in skin irradiated with UV-B radiation [97]. Moreover, a combined regimen of 10% green tea cream and 300 mg of oral green tea supplement twice daily significantly improved skin elasticity in women with moderate PA [104]. Similarly, the daily consumption of a combination of citrus flavonoids and rosemary polyphenols (250 mg) can be considered as an oral photoprotector, as it significantly increased the minimal erythema dose associated with sun exposure after 8 weeks and 12 weeks [105]. The daily consumption of cocoa powder rich in flavonoids, such as epicatechin and catechin, after exposure of the skin to UVR, reduced the associated erythema by 15–25% after 6–12 weeks of treatment, and increased skin hydration and skin thickness while significantly reducing skin roughness and peeling [106].

Cinnamaldehyde is a potent phytochemical antioxidant and the largest component of *Cinnamomum cassia*, a tree which is very similar to cinnamon and is traditionally used in Chinese medicine [107]. The topical application of cinnamaldehyde inhibited the formation of wrinkles caused by repeated UV-B exposure and, at the microscopic level, significantly reduced epidermal hyperplasia [108]. Furthermore, UV-B irradiation induced the expression of MMP13 in mice by destroying the tissue, and the topical application of cinnamaldehyde significantly restored collagen metabolism by decreasing the expression of MMP13. It is believed that human MMP1 in the skin is equivalent to MMP13 in mice [109].

Carotenoids are among the compounds with antioxidant properties most studied in recent years that show beneficial results in skin photoprotection. Carotenoids are a broad group of natural pigments found in various plants, fruits, and vegetables [110,111]. Their relevance to humans lies in their ability to interact with ROS and stabilize them [112]. The most common carotenoids, found in large quantities in regular diets or in the skin, are β-carotenes, α-carotenes, β-cryptoxanthin, lutein, and zeaxanthin [113].

Many in vivo studies have proven the effectiveness of carotenoids as a possible adjuvant treatment for PA [114,115,116,117]. For example, a double-blind, placebo-controlled trial including 60 volunteers with Fitzpatrick skin types II–IV who were orally administered Nutrilite^™^ Multi Carotene supplement, or a placebo daily for 12 weeks, showed that the consumption of carotenoids generated an increase in skin carotenoid levels and, therefore, an increased protection against UVR. Moreover, higher doses of irradiation were needed for the induction of erythema produced by UV-B radiation and pigmentation by UV-A radiation [118]. Similarly, through a randomized clinical trial, Rizwan et al. determined how the carotenoids in tomato paste (rich in lycopene) protected the skin against the effects of UVR in 20 healthy women with phototypes I–II [115]. After ingesting 55 g of tomato paste daily for 12 weeks, the subjects presented a significant decrease in DNA damage and UVR-induced MMP-1 and fibrillin-1 activities, which are all molecular characteristics of PA. Similar results have been observed in other clinical trials [119].

In line with this, nutraceutical supplements rich in minerals, polyphenols, and other antioxidant compounds are potential therapeutic tools against PA. Costa et al. determined the efficacy of a nutraceutical consisting of grape seed extract, acerola extract, lycopene, and Biomarine ComplexT (antioxidant supplement) on the skin of 50 women with phototypes I–III who presented with PA [120]. After the medical assessment, biometric skin analysis, ultrasound, and skin biopsies, a general improvement in the skin was observed in all patients, along with an increase in hydration and density, a decrease in facial seborrhea, and an increase in the production of collagen and elastic fibers in the face and arms. The same group of researchers evaluated the efficacy and safety of a supplement containing vitamin C (27 mg), marine protein (105 mg), zinc (2 mg), grape seed extract (13.75 mg), and tomato extract (14.38 mg) for the treatment of PA in 41 male patients with phototypes I–IV. A significant improvement was observed regarding the presence of erythema, hydration, luminosity, and overall appearance of the skin, accompanied by a decrease in its pH [121]. Similar results have been reported by other authors, who determined the positive role of several oral supplements that combined different phenolic compounds, plant extracts, vitamins, minerals, and proteins with antioxidant properties in individuals with marked PA [122,123,124,125,126,127].

Recent studies have shown that marine-algae-derived agents contain several antioxidant properties beneficial for the skin, and have been found to possess remarkable adaptive mechanisms that enable them to thrive in harsh ecosystems with high exposure to UVR. Likewise, these components that have been developed are capable of counteracting the eliminating effects of UVR, which is believed to be a possible therapeutic target in PA [128]. Sulfated polysaccharides such as carrageenan and fucoidan, mycosporine-like amino acids (MAAs) such as porphyra-334 and shinorin, polyphenols, and carotenoids are among the components that possess photoprotective and antioxidant properties [129].

The significant benefits of these skin components can be seen in their ability to reduce the harmful effects of ROS by downregulating MAPK and NF-κB. As a result, they counteract the subsequent inflammatory, oxidative, and disruptive effects on the skin’s ECM, as mentioned earlier. Among the specific properties in the case of MAAs is their ability to absorb UVR and disperse its energy in the form of heat without the need to produce ROS. Likewise, Ying et al. reported that during an in vivo study conducted on the skin of mice, the use of MAAs resulted in a strengthening of the antioxidant system [130]. This was achieved by countering the inhibition of the decrease in antioxidant enzymes such as SOD, glutathione peroxidase, and catalase. Additionally, a decreased concentration was found in the expression of proinflammatory cytokines such as IL-1beta, IL-6, and IL-10; for this reason, MAAs have been demonstrated to mitigate and prevent skin damage caused by UVR [131,132].

**Table 1:** Summary of clinical evidence of natural antioxidants against photoaging.

Authors	Antioxidant	Methodology	Results
Lauer et al. [81]	Vitamin C	Clinical trial with 33 volunteers who were supplemented with vitamin C or placebo for 4 weeks, using in vivo spectroscopy and placebo control.	Oral administration of vitamin C rapidly enhances skin’s radical-scavenging activity, with higher doses providing greater benefits.
Chiu et al. [104]	Polyphenols	A randomized, double-blind, clinical trial pilot study was conducted to evaluate the effects of a combination regimen of topical and oral green tea supplementation on 40 women who had moderate PA.	There was a significant increase in elastic tissue content in treated specimens at the histological level.
Lephart et al. [100]	Polyphenols	In vitro study on human-skin-equivalent cultures with resveratrol exposure at 1.0% for 24 h.	Resveratrol improves skin aging by stimulating SIRT1, extracellular matrix proteins, and antioxidants while inhibiting biomarkers of inflammation and dermal aging.
Tanaka et al. [108]	Phytochemical	Preclinical study of an in vivo mouse model treated with ointments containing DMSO or CIN and irradiated multiple times with UVB for 10 weeks.	CIN reduces ROS production and repairs DNA damage in vitro. Topical application inhibits wrinkle formation, epidermal hyperplasia, and dermal inflammation. CIN promotes antioxidative processes and prevents UVB-induced collagen degradation.
Rizwan et al. [115]	Carotenoids	Randomized, single-blinded, controlled trial study evaluating 20 women who ingested 55 gr tomato paste (rich in lycopene) daily for 12 weeks.	After supplementing, there was a significant reduction in DNA damage and a decrease in activity of MMP1 and fibrillin-1, all mediated by UVR.
Ying et al. [130]	Marine-algae-derived	In vivo study of a murine model treated with MAA solutions in concentrations of 5, 10, and 20 mg/mL.	The MAA compounds regulated the NF-κB pathway, resulting in increased antioxidant enzymes and decreased proinflammatory cytokines.

Abbreviations: PA: photoaging; SIRT1: Sirtuin-1; CIN: cinnamaldehyde; ROS: reactive oxygen species; MMP1: matrix metalloproteinase 1; UVR: ultraviolet radiation; NFκB: nuclear factor kappa B.

Ozone therapy has also been used for patients with PA. This empirical tool has antioxidant and anti-inflammatory potential because it positively modulates the expression of Nrf2 and enzymes such as SOD and HO-1 [133]. Although the anti-aging and dermato-protective capacity of ozone therapy has been shown in some cases of elderly patients [134,135], further studies are required to establish this method as an adjuvant for the treatment of PA. Similarly, physical activity is known to be a potent antioxidant and anti-inflammatory agent, which makes it one of the potential adjuvants in therapy against CA and PA. The mechanisms underlying its possible role in dermatological clinical practice lie in (a) the activation of skeletal muscle AMP-activated protein kinase and the subsequent expression of IL-15 [136], and (b) mitochondrial dysfunction and OS [137]. However, clinical studies determining the relationship between exercise and PA are scarce.

## 7. Conclusions

PA is a pathological process that, unlike CA, occurs because of external factors such as skin exposure to UVR, particularly UV-B. UVR is capable of disrupting the physiological functioning of the skin, either by affecting the production of collagen in fibroblasts, increasing the activity of matrix proteins such as MMPs, increasing oxidative and proinflammatory processes in the tissue, or damaging the DNA. All these changes translate into morphological alterations in the skin similar to those caused by CA, such as wrinkles, decreased skin elasticity, and hyperpigmentation.

The scientific community has focused on exploring ways to modulate the body’s antioxidant machinery to counteract PA through the administration of exogenous products with antioxidant properties, such as medicinal herbs, nutraceuticals, and fruits. This has been supported by high-quality clinical evidence, which shows that the polyphenols, vitamins, minerals, and carotenes in these foods or supplements can counteract the pathological processes of PA and, thereby, improve the appearance of the skin. These antioxidants are considered the cornerstone in PA treatment due to their action in protecting against genome damage by preventing DNA and other molecules from absorbing UVR. Additionally, the antioxidants mentioned above can also inhibit or counteract ROS formation and act as ECM regulators because they modulate MMP activity and collagen production, both mechanisms that are implicated in the PA pathophysiology. However, the primary prevention approach based on the use of sunscreens and other skin photoprotection elements should not be disregarded.

Notwithstanding, photofragmentation, electron transfer reactions, and/or photoisomerization are processes that can cause unfavorable reactions between formulation components and produce reactive byproducts that may limit their application. The incidence of such deleterious effects is unknown, so more studies are needed to establish a solution for this problem. However, generally the benefits outweigh the risks in this type of photoprotective management. Other factors that could limit the use of this formulation are specific for each case; for example, allergies or hypersensitivity reactions due to the components of the formulation could limit use.

In addition, more research is required to assess the role of natural antioxidants against PA for them to be approved and added to the standard approach against this pathology. Currently, several clinical trials are assessing the use and safety of oral nutritional supplements, fruits, and other phytochemicals in the treatment of PA [138,139,140,141,142].

## Data Availability

Not applicable.
